# Biotinidase is a Novel Marker for Papillary Thyroid Cancer Aggressiveness

**DOI:** 10.1371/journal.pone.0040956

**Published:** 2012-07-23

**Authors:** Anthony K.-C. So, Jatinder Kaur, Ipshita Kak, Jasmeet Assi, Christina MacMillan, Ranju Ralhan, Paul G. Walfish

**Affiliations:** 1 Alex and Simona Shnaider Laboratory in Molecular Oncology, Department of Pathology and Laboratory Medicine, Mount Sinai Hospital, Joseph & Wolf Lebovic Health Complex, Toronto, Ontario, Canada; 2 Joseph and Mildred Sonshine Family Centre for Head and Neck Diseases, Mount Sinai Hospital, Toronto, Ontario, Canada; 3 Department of Pathology and Laboratory Medicine, Mount Sinai Hospital, Joseph & Wolf Lebovic Health Complex, Toronto, Ontario, Canada; 4 Department of Otolaryngology–Head and Neck Surgery, Mount Sinai Hospital, Toronto, Ontario, Canada; 5 Department of Otolaryngology–Head and Neck Surgery, University of Toronto, Toronto, Ontario, Canada; University of Texas MD Anderson Cancer Center, United States of America

## Abstract

Biotinidase was identified in secretome analysis of thyroid cancer cell lines using proteomics. The goal of the current study was to analyze the expression of biotinidase in thyroid cancer tissues and fine needle aspiration (FNA) samples to evaluate its diagnostic and prognostic potential in thyroid cancer. Immunohistochemical analysis of biotinidase was carried out in 129 papillary thyroid cancer (PTC, 34 benign thyroid tissues and 43 FNA samples and correlated with patients’ prognosis. Overall biotinidase expression was decreased in PTC compared to benign nodules (p = 0.001). Comparison of aggressive and non-aggressive PTC showed decrease in overall biotinidase expression in the former (p = 0.001). Loss of overall biotinidase expression was associated with poor disease free survival (p = 0.019, Hazards ratio (HR) = 3.1). We examined the effect of subcellular compartmentalization of nuclear and cytoplasmic biotinidase on patient survival. Decreased nuclear expression of biotinidase was observed in PTC as compared to benign tissues (p<0.001). Upon stratification within PTC, nuclear expression was reduced in aggressive as compared to non-aggressive tumors (p<0.001). Kaplan-Meier survival analysis showed significant association of loss of nuclear biotinidase expression with reduced disease free survival (p = 0.014, HR = 5.4). Cytoplasmic biotinidase expression was reduced in aggressive thyroid cancers in comparison with non-aggressive tumors (p = 0.002, Odds ratio (OR) = 0.29) which was evident by its significant association with advanced T stage (p = 0.003, OR = 0.28), nodal metastasis (p<0.001, OR = 0.16), advanced TNM stage (p<0.001, OR = 0.21) and extrathyroidal extension (p = 0.001, OR = 0.23). However, in multivariate analysis extrathyroidal extension emerged as the most significant prognostic marker for aggressive thyroid carcinomas (p = 0.015, HR = 12.8). In conclusion, loss of overall biotinidase expression is a novel marker for thyroid cancer aggressiveness.

## Introduction

Thyroid cancer is the most common malignant endocrine tumor and accounts for >90% of cancers of the endocrine glands, with an estimated annual incidence of 122,803 cases worldwide [1]. Most thyroid cancers have an excellent prognosis; both papillary and follicular thyroid cancers have about 85% to 90% cure rates, if detected early and treated appropriately. However, a small percentage is in fact aggressive and may develop distant metastasis leading to higher mortality [2]. In view of the more rapid increase in the incidence of thyroid cancer than any other solid tumor (about 3 per 100,000 people per year), anaplastic thyroid cancer and other aggressive variants pose a major challenge to oncologists [3]. Anaplastic thyroid cancer accounts for less than 2% of all thyroid cancers, yet, it causes up to 50% of deaths from this malignancy annually; 90% of anaplastic thyroid cancer patients die within 6 months of diagnosis (the median survival rate is 4 months) [3], [4]. Anaplastic thyroid cancer and aggressive variants of papillary thyroid cancer, follicular and metastatic thyroid cancers have high risk of recurrence, shortened disease free survival and death within 5 to 10 years [3]. Furthermore, anaplastic thyroid cancer is highly resistant to conventional cancer therapy. Consequently, anaplastic thyroid cancer and aggressive variants are the source of significant morbidity and mortality in a disease that otherwise boasts of an excellent prognosis. The key to narrowing the wide gap of prognosis between aggressive and non-aggressive variants is to detect the instigating factor(s) that are responsible for aggressive behavior. Currently, there is a lack of molecular markers to predict the aggressiveness of thyroid cancer.

At present, fine-needle aspiration (FNA) is the most commonly used pre-operative technique for diagnosis of thyroid nodules >1 cm in size. However, even the use of ultrasound-guided FNA is often beset with inconclusive biopsy results (10–20% of all cases) [5]. These patients undergo subsequent thyroidectomy, an invasive procedure that is often unnecessary as the majority of the suspected lesions are benign (>80%) [6]. In addition, recurrent cases require additional treatment in the form of surgery or radioactive iodine ablation that further compromises their quality of life. The time is ripe for early identification of aggressive cases through biomarker(s) detection and categorization of high risk patients. Hence, there is an urgent need for identifying biomarkers that can be used as an adjunct to FNA to distinguish benign thyroid nodules from malignant tumors (especially for more accurate diagnosis of indeterminate cases) and aid discrimination of aggressive thyroid cancers from their non-aggressive counterparts post-surgery to better define patient management.

In search of new cancer biomarkers for this malignancy, we analyzed the secretome from thyroid cancer cell lines to identify cancer-relevant secreted proteins that can serve as potential biomarkers [7]. One of the candidate proteins identified in our study was biotinidase, an enzyme that catalyzes the hydrolysis of biocytin, the product of biotin-dependent carboxylase degradation, to biotin and lysine. Biotin deficiency may lead to the decreased activity of holocarboxylase synthetase, an enzyme which mediates the binding of biotin to histones [8], a crucial component of epigenetic events that regulate chromatin structures and gene function. Low level of biotinidase was observed in aggressive anaplastic derived cell line (CAL-62) as compared to the non-aggressive papillary derived thyroid cancer cell line (TPC-1). The clinical relevance was suggested by demonstrating reduced levels of biotinidase in aggressive thyroid cancer patients’ sera as compared to the non-aggressive and benign patients’ sera by western blotting [7]. In the current study, our main objective was to determine the clinical significance of biotinidase as a marker to distinguish benign thyroid and malignant tumors as well as to stratify aggressive and non-aggressive PTC that could serve as a potential tool for improved management of this malignancy.

## Materials and Methods

### Clinicopathological Characteristics of Patients and Tissue Specimens

This study was approved by the Research Ethics Board of Mount Sinai Hospital, Toronto, Ontario, Canada. Archived formalin-fixed paraffin-embedded tissue blocks from the Mount Sinai Hospital Tumor Bank were retrieved, reviewed by the pathologist (CM), and cut to provide 5 µm-thick sections for immunohistochemical staining. Diagnoses were derived from histopathological analyses and clinical examination. Benign cases included multinodular goiters, hyperplastic nodules, and follicular adenomas, whereas all non-aggressive and aggressive tumors examined here were well-differentiated papillary thyroid carcinomas which included the following variants: classic, follicular, oncocytic, diffuse sclerosing, and tall cell. Defining features of tumor aggressiveness were TNM stage IV classification, distant metastasis, perineural invasion, and disease recurrence or persistence. Additional consideration was given to the following potential risk factors of aggressiveness including: TNM stage III, vascular invasion, extrathyroidal extension, lymph node metastasis, and non-classical papillary thyroid cancer variant type (especially the presence of three or more of these factors in the cases examined).

Based on these criteria, the 163 tissue samples examined in this study were categorized as 34 benign (median age: 51 years; range: 16 to 76 years), 81 non-aggressive (median age: 44 years; range: 23 to 71 years), and 48 aggressive (median age: 52 years; range: 21 to 86 years) tumors.

Formalin-fixed paraffin-embedded cell blocks from fine-needle aspiration preparations from 43 patients were similarly obtained from the Mount Sinai Hospital Tumor Bank and each sample diagnosis was confirmed by histological examination of the respective thyroidectomy specimen (CM). Accordingly, the FNA samples were categorized as 23 benign, 13 non-aggressive, and 7 aggressive cases.

The patient follow up data were retrieved from the clinical database to correlate the protein expression in tumors with clinical outcome for evaluation of the prognostic relevance of biotinidase. The patients were followed up for a maximum period of 19.5 years.

### Immunohistochemical Analysis of Biotinidase Expression in Thyroid Carcinomas and FNA Samples

Slides were immunostained as described previously [9], using α-biotinidase K-17 rabbit polyclonal antibody (Santa Cruz Biotechnology; 1∶100 dilution) raised against a peptide mapping to an internal region of human biotinidase. The antigen retrieval was performed with Tris-EDTA buffer (10 mM Tris base and 1 mM EDTA with 0.05% Tween 20, pH 9.0) in a microwave for 20 min. In the negative control tissue sections, the primary antibody was replaced by isotype-specific non-immune mouse IgG. The sections were evaluated by light microscopic examination. Images were captured using the Visiopharm Integrator System (Horsholm, Denmark).

### Evaluation of Immunohistochemical Staining

Immunopositive staining was evaluated in five areas of the tissue sections as described [9]. Sections were scored as positive if epithelial cells showed immunopositivity in the cytoplasm, and/or nucleus when observed by two evaluators who were blinded to the clinical outcome. These sections were scored as follows: 0, <10% cells; 1, 10–30% cells; 2, 30–50% cells; 3, 50–70% cells; and 4, >70% cells showed immunoreactivity. Sections were also scored semi-quantitatively on the basis of intensity as follows: 0, none; 1, mild; 2, moderate; and 3, intense. Finally, a total score (ranging from 0 to 7) was obtained by adding the scores of percentage positivity and intensity for each of the benign thyroid and malignant tumor tissue sections. The immunohistochemical data were subjected to statistical analysis.

### Follow-up Study

Of the 129 thyroid cancer cases, follow-up data were available for 116 (90%) patients, while 13 patients (10%) were lost to follow-up. Thyroid cancer patients were monitored for a maximum period of 19.5 years (Range 2–234 months; mean 43 months and median 29 months). Recurrence with or without metastases was observed in 19 of 116 (16.4%) patients monitored during the follow-up. Ninety seven patients who did not show recurrence were alive until the end of the follow-up period. Disease-free survival was expressed as the number of months from the date of surgery to recurrence or till the last possible follow up in case of patients who are disease free.

**Table 1:** Correlation of biotinidase expression with clinico-pathological parameters of thyroid cancer patients.

Clinico-pathologic parameters	Total N	Cyt Positiven (%)	Cyt P value	Cyt OR (95% C.I)	Nuc Positiven (%)	Nuc P value	Nuc OR (95% C.I)	Overall Positiven (%)	overall P value	Overall OR (95% C.I)
**Benign**	**34**	**9 (26.5)**	**<0.001**	**5.8 (2.47–13.41)**	**33 (97.1)**	**<0.001**	**0.02 (0.00–0.12)**	**33 (97.1)**	**0.001**	**14.3 (1.9–108.3)**
**Cancer**	**129**	**87 (67.4)**			**45 (34.9)**			**90 (69.8)**		
**Age (Years) <45**	59	38 (64.4)	0.573	1.29 (0.62–2.69)	24 (40.7)	0.266	0.63 (0.30–1.29)	40 (67.8)	0.703	0.84 (0.40–1.79)
**≥45**	70	49 (70)			21 (30)			50 (71.4)		
**Gender Female**	105	72 (68.6)	0.631	0.76 (0.30–1.92)	37 (35.2)	1.000	0.92 (0.36–2.35)	74 (70.5)	0.806	1.19 (0.46–3.08)
**Male**	24	15 (62.5)			8 (33.3)			16 (66.7)		
**Tumor T T1+T2**	96	72 (75)	**0.003**	0.28 (0.12–0.63)	42 (43.8)	**<0.001**	0.13 (0.04–0.45)	75 (78.1)	**0.001**	4.29 (1.85–9.92)
**T3+T4**	33	15 (45.5)			3 (9.1)			15 (45.5)		
**Nodal Status N0**	80	66 (82.5)	**<0.001**	0.16 (0.07–0.36)	40 (50)	**<0.001**	0.11 (0.04–0.32)	68 (85)	**<0.001**	6.96 (3.02–15.99)
**N+**	49	21 (42.9)			5 (10.2)			22 (44.9)		
**Stage I+II**	97	74 (76.3)	**<0.001**	0.21 (0.09–0.50)	41 (42.3)	**0.002**	0.20 (0.06–0.60)	76(78.4)	**0.001**	4.65 (1.99–10.88)
**III+IV**	32	13 (40.6)			4 (12.5)			14(43.8)		
**Type Non-agg**	81	63 (77.8)	**0.002**	0.29 (0.13–0.62)	38 (46.9)	**<0.001**	0.19 (0.08–0.48)	65 (80.2)	**0.001**	3.74 (1.70–8.21)
**Agg**	48	24 (50)			7 (14.6)			25 (52.1)		
**Multifocal No**	41	31 (75.6)	0.227	0.57 (0.25–1.30)	18 (43.9)	0.167	0.57 (0.26–1.22)	31 (75.6)	0.411	1.52 (0.66–3.53)
**Yes**	88	56 (63.6)			27 (30.7)			59 (67)		
**Microcarcinoma/Occult No**	43	25 (58.1)	0.117	1.86 (0.86–4.01)	14 (32.6)	0.845	1.17 (0.54–2.53)	26(60.5)	0.110	0.53 (0.24–1.15)
**Yes**	86	62 (72.1)			31 (36)			64 (74.4)		
**Encapsulated No**	64	40 (62.5)	0.263	1.57 (0.75–3.29)	17 (26.6)	0.065	2.09 (1–4.39)	41 (64.1)	0.183	0.58 (0.27–1.25)
**Yes**	65	47 (72.3)			28 (43.1)			49 (75.4)		
**Perineural Invasion No**	126	84 (66.7)	0.550	0.67 (0.59–0.75)	44 (34.9)	1.000	0.93 (0.08–10.57)	87 (69)	0.553	0.69 (0.61–.078)
**Yes**	3	3 (100)			1 (33.3)			3 (100)		
**Extra Thyroidal Extension No**	94	72 (76.6)	**0.001**	0.23 (0.1–0.52)	40 (42.6)	**0.003**	0.23 (0.08–0.63)	75 (79.8)	**<0.001**	5.27 (2.28–2.16)
**Yes**	35	15 (42.9)			5 (14.3)		**0.02 (0.00–0.12)**	15 (42.9)		

N = number of cases, Cyt = Cytoplasmic staining, Nuc = Nuclear staining, OR = Odds ratio,

C.I = Confidence interval, Non-agg = Non-aggressive, Agg = Aggressive

**Figure 1:**
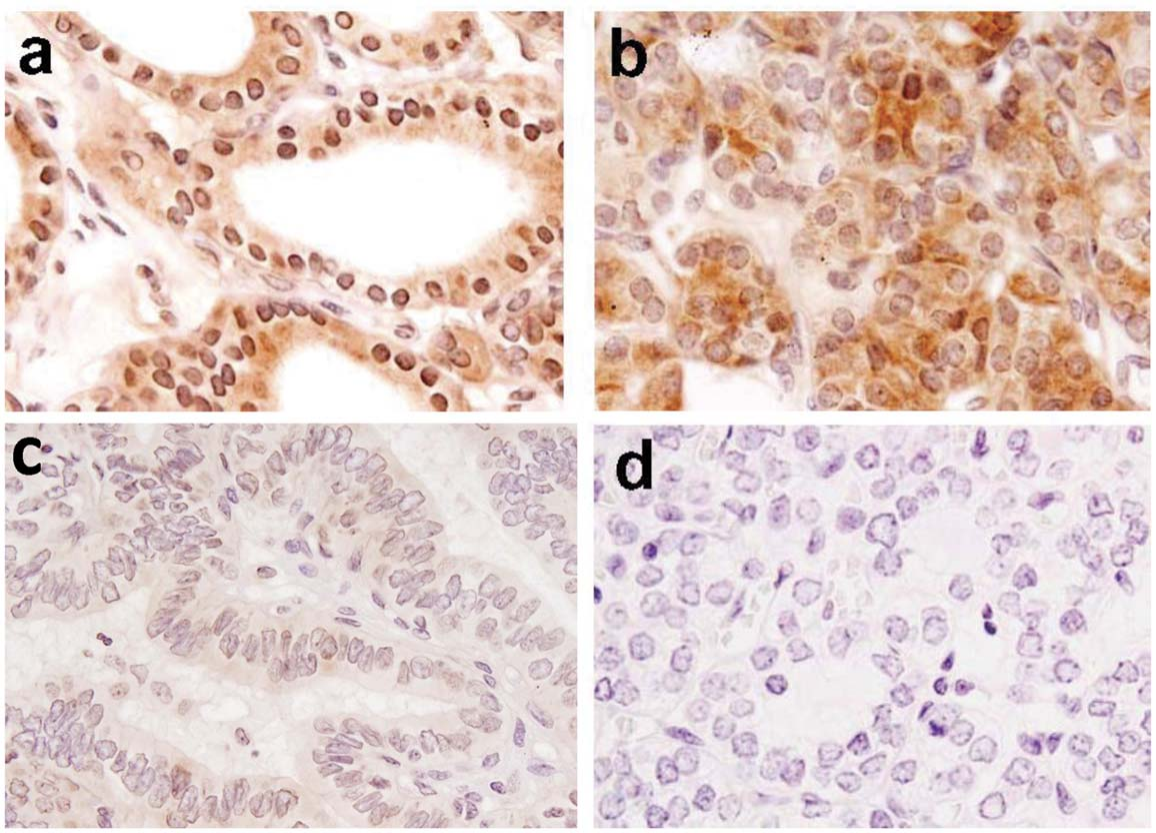
Immunohistochemical analysis of biotinidase in thyroid tissues. Paraffin embedded sections of benign thyroid nodules and malignant tumors were stained using anti-biotinidase polyclonal antibody as described in the Methods section: a) Benign tissue section showing nuclear and overall biotinidase immunostaining; b) Papillary non-aggressive thyroid cancer section illustrating reduction in nuclear staining and increase of cytoplasmic biotinidase immunostaining in the tumor cells; c) Papillary aggressive thyroid cancer section showing reduced overall (nuclear and cytoplasmic) biotinidase immunostaining; d) Thyroid cancer section used as a negative control, showing no immunoreactivity in cells (a–d, original magnification x 400).

### Statistical Analysis

The immunohistochemical data were subjected to statistical analysis using SPSS 17.0 software (SPSS Inc., Chicago, IL). Scatter plots were used to determine the distribution of cytoplasmic, nuclear and overall biotinidase expression in benign, non-aggressive and aggressive thyroid cancers. Sensitivity and specificity were calculated and quantified using receiver operating characteristic (ROC) analysis. Based on the optimal sensitivity and specificity as revealed by ROC analysis, cut-offs were generated for biotinidase protein expression. For overall biotinidase expression, a cut-off value of ≥3.6 was defined as positive immunoreactivity for statistical analysis. A cut-off value of ≥2 and ≥4 was defined as positive criterion for individual nuclear and cytoplasmic biotinidase immunopositivity respectively for detailed statistical examination. The relationships between biotinidase expression and clinicopathological parameters were tested using Chi-Square and Fischer’s exact test. Two-sided p values were calculated and p<0.05 was considered to be significant. Prognostic significance of overall, nuclear and cytoplasmic biotinidase expression was assessed by Kaplan-Meier survival and multivariate Cox-proportional Hazards regression analysis.

**Figure 2:**
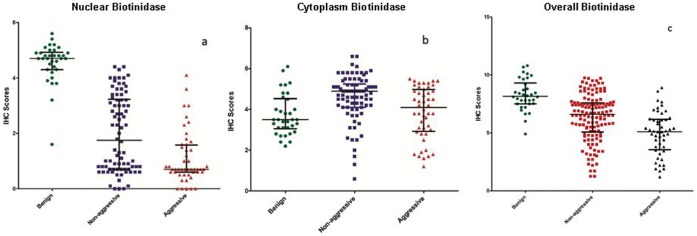
Scatter plot analysis of biotinidase nuclear, cytoplasmic and overall expression. Scatter plots showing distribution of total immunostaining scores determined by IHC of tissue sections of benign (n = 34), non-aggressive (n = 81) and aggressive (n = 48) thyroid cancer tissues. The vertical axis gives the total immunohistochemical score as described in the Methods. a) Decreased nuclear accumulation of biotinidase was observed in aggressive and non-aggressive thyroid cancers analyzed. Scatter plot shows the reduction in nuclear biotinidase immunostaining with increasing tumor aggressiveness; b) Increased cytoplasmic accumulation of biotinidase was observed in the thyroid cancer analyzed with reduction in expression in aggressive compared to non-aggressive PTC; c) Scatter plot showed reduced overall biotinidase immunostaining with increasing tumor aggressiveness.

**Table 2:** Receiver operating characteristic curve analysis of biotinidase expression in benign vs malignant and aggressive vs non-aggressive thyroid cancer.

Benign vs Malignant Thyroid						
Biotinidase	AUC	Sensitivity (%)	Specificity (%)	NPV (%)	PPV (%)	Asymptotic significance
Cytoplasmic	0.662	66.9	74.0	36.2	90.8	<0.001
Nuclear loss	0.972	97.1	65.0	1.1	58.2	<0.001
Overall loss	0.816	79.4	69.2	5.3	59.7	<0.001
**Aggressive vs Non-aggressive PTC**						
**Biotinidase**	**AUC**	**Sensitivity (%)**	**Specificity (%)**	**NPV (%)**	**PPV (%)**	**Asymptotic significance**
Cytoplasmic	0.347	76.2	49.0	71.9	54.4	0.003
Nuclear loss	0.696	46.4	85.7	84.8	48.3	<0.001
Overall loss	0.715	42.9	89.8	71.7	56.1	<0.001

AUC, area under the curve; NPV, negative predictive value; PPV, positive predictive value.

**Figure 3:**
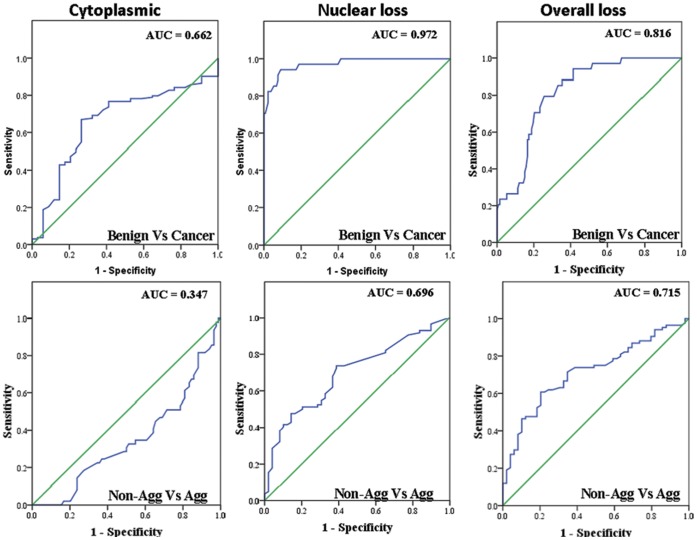
ROC curves analyses of cytoplasmic, nuclear and overall biotinidase expression in thyroid cancers. The vertical axis of each curve indicates sensitivity and the horizontal axis indicates the 1-specificity. The sensitivity, specificity, and AUC values are summarized in Tables 2a and 2b.

## Results

### Immunohistochemical Analysis of Biotinidase Expression in Thyroid Tissues

Of the 34 benign tissues analyzed, 33 tissues (97.1%) showed overall as well as nuclear accumulation of biotinidase protein (Table 1, Figure 1a). Cytoplasmic expression of biotinidase was observed in 9 (26.5%) benign tissues (Table 1). Among the malignant thyroid tissues, 39/129 (30.2%) demonstrated loss of overall (both in nucleus and cytoplasm) biotinidase expression. Upon stratification into nuclear and cytoplasmic subcellular compartments, 84/129 cases (65.1%) showed loss of nuclear biotinidase and 87/129 (67.4%) showed increased cytoplasmic expression in tumor cells (Table 1, Figure 1b). Notably, loss of overall as well as nuclear biotinidase and increase in cytoplasmic biotinidase expression was significant in malignant tumors in comparison to benign nodules (p = 0.001, p<0.001, p<0.001 respectively Table 1). In aggressive thyroid cancer, there was significant reduction in overall biotinidase expression (p = 0.001) at both nuclear (p<0.001) and cytoplasmic (p = 0.002) levels as compared to non-aggressive thyroid tumors (Table 1, Figures 1c and 2). No immunostaining was observed in tissue sections used as negative controls where the primary antibody was replaced by isotype specific IgG (Figure 1d).

**Figure 4:**
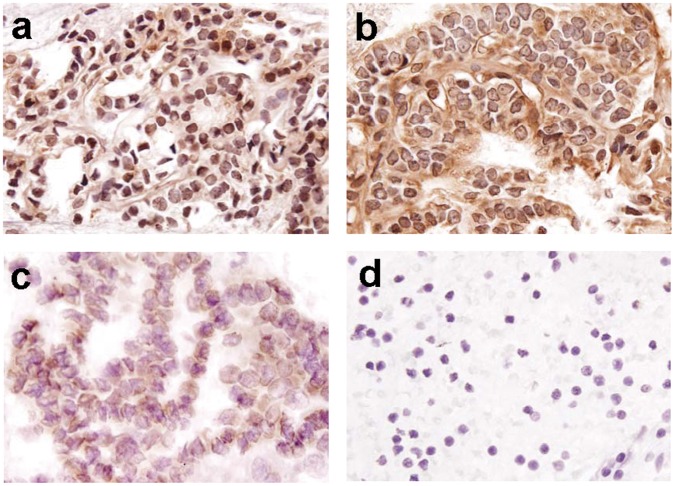
Biotinidase expression in thyroid FNA samples. FNA specimens from benign (Panel a), non-aggressive papillary thyroid cancer (Panel b), and aggressive papillary thyroid cancer (Panel c) cases were immunostained with 1∶100 α-biotinidase K-17 rabbit polyclonal antibody. FNA specimen used as a negative control shows no immunoreactivity in cells (Panel d). Photomicrographs show a pronounced decrease in nuclear biotinidase expression in more aggressive thyroid cancer cases and are presented at 400× original magnification.

### Reduced Overall Biotinidase Expression Correlated with Tumor Aggressiveness

The reduction in overall biotinidase expression significantly correlated with advanced T stage (p = 0.001, OR = 4.29), nodal metastasis (p<0.001, OR = 6.96), Stage III+IV tumors (p = 0.001, OR = 4.65), and extrathyroidal extension (p<0.001, OR = 5.27). Furthermore, loss of cytoplasmic and nuclear biotinidase individually also showed significant association with advanced T stage (p = 0.003, p<0.001 respectively), nodal metastasis (p<0.001, p<0.001 respectively), stage III+IV tumors (p<0.001, p = 0.002 respectively), and extrathyroidal extension (p = 0.001, p = 0.003 respectively; Table 1). These findings suggest that the loss of biotinidase at both cytoplasmic (p = 0.002, Table 1) and nuclear (p<0.001, Table 1) level is associated with aggressive phenotype of thyroid cancer. The association of reduction in nuclear and cytoplasmic biotinidase to these clinical parameters provides additional credibility to the loss of overall biotinidase correlating significantly with tumor aggressiveness (p = 0.001, Table 1).

**Figure 5:**
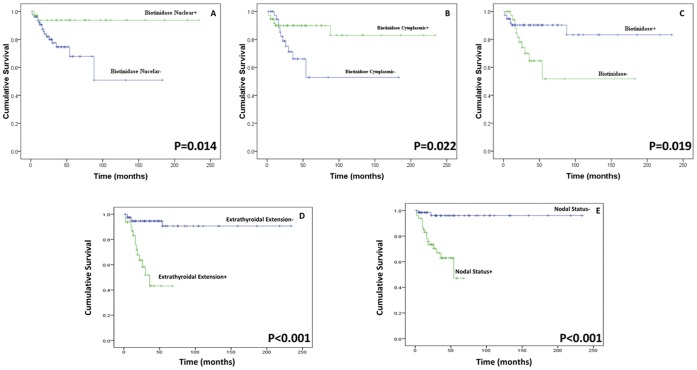
Kaplan–Meier estimation of cumulative proportion of disease-free survival in thyroid cancer patients. Disease free survival curves showing biotinidase expression in (a) nucleus [median disease-free survival 116 months]; (b) cytoplasm [median disease-free survival 111 months]; (c) and overall (nucleus and cytoplasm) [median disease-free survival 109 months]. Disease free survival curves showing (d) extrathyroidal extension [median disease-free survival 36 months], and (e) nodal status [median disease-free survival 54 months].

**Table 3:** Multivariate analysis for correlation of biotinidase expression with disease free survival.

				95% CI for HR	
Univariate	Parameter	P value	Hazards ratio (HR)	Lower	Upper
	Age	0.595	0.8	0.3	1.9
	Gender	0.136	2.1	0.8	5.5
	Tumor stage	**<0.001**	2.2	1.5	3.2
	Overall stage	0.140	1.3	0.9	1.8
	Histology type	0.676	1.1	0.8	1.4
	Histology grade	0.374	23.9	0.02	26100.0
	Nodal status	**0.001**	12.2	2.8	53.8
	Extrathyroidal extension	**<0.001**	9.0	3.1	26.4
	Biotinidase Nuclear loss	**0.014**	5.4	1.2	24.1
	Biotinidase Cytoplasmic loss	0.022	2.8	1.1	7.2
	Biotinidase Overall loss	**0.019**	3.1	1.2	7.8
**Multivariate**					
	Extrathyroidal extension	**0.015**	4.1	1.3	12.8
	Nodal status	**0.035**	5.6	1.1	27.8

### Potential of Biotinidase as a Biomarker for Thyroid Cancer

Receiver operating characteristic curve analysis was used to determine the potential of biotinidase expression as a biomarker to distinguish benign nodules and malignant tumors (Table 2). Loss of overall and nuclear as well as increased cytoplasmic expression of biotinidase distinguished benign tissues from malignant tumors with AUC values of 0.816, 0.972 and 0.662 respectively (Figure 3, Table 2). Upon stratification of cancer into aggressive and non-aggressive tumors, reduced overall, nuclear and cytoplasmic expression of biotinidase was observed in aggressive thyroid cancers with AUC values of 0.715, 0.696 and 0.347 respectively. (Figure 3, Table 2).

### Detection of Biotinidase Expression in Fine Needle Aspirates of Thyroid Cancer Patients Samples

We analyzed the expression of biotinidase in 23 benign and 20 malignant thyroid FNA samples (Figure 4). Similar pattern of expression was observed in FNA sections as observed in the surgically resected thyroid tissue samples. Significant reduction in overall biotinidase expression was observed upon comparing benign FNA sections (23/23, 100%) to malignant sections (13/20, 65%) (p = 0.002). All the benign FNA sections showed nuclear expression (23/23, 100%) of biotinidase protein as compared to thyroid cancer sections (13/20, 65%; p = 0.002). Cytoplasmic expression of biotinidase was observed in 6/23 (26.1%) benign cases as compared to 2/20 (10%) in thyroid cancer cases (p = 0.25, OR = 0.315, 95% C.I. = 0.056−1.78). Upon comparison of aggressive and non-aggressive thyroid cancers, overall biotinidase expression reduced in the former (5/7, 71.4%) compared to the latter (2/13, 15.4%, p = 0.022). Moreover, significantly reduced nuclear expression was observed in aggressive (5/7, 71.4%) thyroid cancer cases as compared to non-aggressive cases (2/13, 15.4%, p = 0.022, OR = 0.073, 95% C.I. = 0.008−0.674). Similarly, reduced cytoplasmic expression of biotinidase was observed in aggressive (7/7, 100%) thyroid cancer cases as compared to the non-aggressive cases (11/13, 84.6%, p = 0.521).

### Overall Biotinidase Loss as a Predictor of Disease Progression and Prognosis

The follow-up data of 116 thyroid cancer patients for up to 19.5 years were used to assess the prognostic relevance of biotinidase for predicting recurrence in these patients after completion of primary treatment. Kaplan-Meier survival analysis showed significantly reduced disease free survival in patients with decreased expression of biotinidase in nucleus (p = 0.014, HR = 5.4, 95% C.I. = 1.2–24.1; median survival 116 months, Figure 5a), cytoplasm (p = 0.022, HR = 2.8, 95% C.I. = 1.1–7.2; median survival 111 months, Figure 5b) and overall (p = 0.019, HR = 3.1, 95% C.I. = 1.2–7.8; median survival 109 months, Figure 5c). Kaplan-Meier survival analysis showed significantly reduced disease free survival in patients with extrathyroidal extension (p<0.001, HR = 9.0, 95% C.I. = 3.1–26.4); median survival 36 months, Figure 5d), and nodal status (p<0.001, HR = 12.2, 95% C.I. = 2.8–53.8; median survival 54 months, Figure 5e). Cox regression analysis (multivariate) was carried out to determine the prognostic potential of nuclear and cytoplasmic biotinidase, individually and in conjunction, for thyroid cancer patients in comparison with age, gender, t-staging, nodal status, overall stage, histology type, histology grade, and extrathyroidal extension (Table 3). Extrathyroidal extension and nodal status emerged more significant than biotinidase as markers for poor prognosis (p = 0.015, HR = 4.1, 95% C.I. = 1.3–12.8, and p = 0.035, HR = 5.6, 95% C.I. = 1.1–27.8 respectively).

## Discussion

There is an unmet need to identify novel biomarker(s) that can not only help distinguish benign thyroid from malignant tumors but also have the ability to discriminate between aggressive and non-aggressive thyroid cancers. The findings of our study suggest that biotinidase satisfies both these criteria. In addition, our study demonstrates biotinidase levels are decreased in aggressive thyroid carcinomas and suggests its potential to serve as a marker for tumor aggressiveness. This novel use of biotinidase underscores its potential to serve as a tool to identify aggressive thyroid cancers in early stages for more focused therapy. Recently Kang *et al* [10] observed lowered biotinidase levels in plasma of breast cancer patients by proteome analysis and suggested biotinidase as a potential serological biomarker for the diagnosis of breast cancer. In another study, Huang *et al* [11] identified a gene expression profile consisting of 11 genes that could predict pelvic lymph node metastasis in cervical carcinoma using oligonucleotide microarray. Intriguingly, one of the genes in their panel was biotinidase, down regulated in pelvic lymph node metastasis, akin to our findings in thyroid cancer. These studies support our findings that biotinidase is significantly reduced in aggressive thyroid cancer. The corroborative evidence from aggressive phenotypes of cancers other than thyroid further eludes to a potential role that biotinidase might play in the general mechanism of cancer aggressiveness.

FNA is an essential tool for the management of thyroid nodules and FNA of all thyroid nodules >1 cm has been recommended [12], [13]. FNA provides a safer alternative to thyroidectomy as a diagnostic tool, as only 5% of thyroid nodules are malignant [14]. Expression of overall and nuclear biotinidase in benign FNA samples and its loss in malignant cases, specifically in aggressive thyroid cancers, corresponds to the pattern observed in thyroid cancer tissues. Thus biotinidase may have applicability as a potential diagnostic marker for FNA samples with inconclusive diagnosis and can potentially reduce unnecessary thyroid resections. This could significantly diminish the morbidity associated with unwarranted surgery and provide a more systematic approach to the recognition and management of high risk patients. However, these findings require validation in a larger cohort of patients to delineate its potential as an adjunct to cytological or histological findings. The functional significance of biotinidase in development and/or progression of cancer remain unknown. Nevertheless, the findings of our study indicate decrease in biotinidase levels in aggressive thyroid carcinomas.

Classically age, gender, tumor stage, extrathyroidal extension (spread outside the thyroid capsule), nodal status, histology type, histology grade all have prognostic significance and were observed to be associated with biotinidase expression in our study. To determine independent prognostic significance for biotinidase, all these conventional markers of poor prognosis for thyroid cancer were incorporated into a multivariate model and the additional significance of biotinidase was assessed. However, in multivariate analysis extrathyroidal extension and nodal status emerged more significant than biotinidase as markers for poor prognosis (p = 0.015, HR = 4.1, 95% C.I. = 1.3−12.8, and p = 0.035, HR = 5.6, 95% C.I. = 1.1−27.8 respectively). Given the association of biotinidase with conventional markers of poor prognosis, biotinidase did not have independent prognostic significance in this study cohort. Nevertheless, our findings are of significance in view of the limited studies on biotinidase in human cancers; importantly these few reports corroborate and support our observations.

The role biotinidase plays in cancer aggressiveness remains to be elucidated. A probable hypothesis would center on the role of biotin as a co-factor for a plethora of enzymes responsible for chromatin structure and stability. Biotinidase cleaves biocytin thereby making free biotin readily available. Loss of this enzyme would subsequently cause a biotin deficient state which would in turn affect histone biotinylation in chromatin remodeling. It is known that biotinylation of K12 in histone H4 is important for repair of DNA and heterochromatin structures as well as repression of genes and transposons to maintain genomic stability and reduce cancer risk in human cells and *Drosophila melanogaster* [8]. It could be speculated that biotin deficiency might lead to critical epigenetic alterations in cancer attributing an aggressive phenotype to it in the process. Whether the loss of biotinidase plays a functional role or is associated with cancer aggressiveness remains to be addressed in future studies. Nevertheless, our findings are useful and will be applicable for clinical use alone or in combination with other biomarker(s) in diagnosis and/or prognosis of aggressive thyroid cancers.

BRAF(V600E) is considered a negative prognostic marker in PTC and might have been a confounding prognostic factor in our analysis. One of the limitations of our study is the non-availability of BRAF(V600E) mutation data in our cohort of thyroid cancer patients. The majority of PTCs are initiated by genetic events involving mutation of BRAF or RAS and translocations producing RET/PTC oncogenes [15]. BRAFV600E mutation is found in approximately 40% of PTC and in more than 50% poorly differentiated thyroid cancers [16], [17]. The constitutive activation of BRAF caused by BRAF(V600E) mutation leads to activation of the RET/RAS/BRAF/MAPK signal transduction pathway and plays an important role in cell proliferation by regulating cyclin D and p27 [18]. This mutation is also associated with decreased expression of mRNAs for the sodium iodide symporter and the TSH receptor, markers of thyroid differentiation [19]. BRAF mutation is associated with progression of PTC to poorly differentiated thyroid carcinomas due to increased sensitivity to TGFβ-induced epithelial-mesenchymal transition (EMT) [20], and with vascular endothelial growth factor (VEGF) overexpression and associated higher risk of metastasis, recurrence and shorter disease free survival [21], [22]. A high percentage of BRAF(V600E) alleles defines a PTC molecular subtype and predicts a poorer disease outcome [23], [24] and analysis of BRAF mutations by pyrosequencing has recently been demonstrated to be useful to refine the risk stratification of PTC patients [25]. However, unexplained conflicting results are also reported in the literature. A recent study showed BRAF(V600E) is common in Finnish patients with low-risk PTC but does not predict recurrence after long-term follow-up of initial treatment with total thyroidectomy and radioiodine remnant ablation [26]. In another recent study involving 4585 consecutive patients who were found to have malignant or indeterminate thyroid nodules by ultrasonography, BRAF(V600E) mutation analysis using three independent molecular assays in FNA cytology specimens did not show any significant correlation with multifocality, extrathyroidal extension, and lymph node metastasis [27]. The exploration of the relationship between reduced biotinidase, BRAF (V600E) mutation and aggressive thyroid cancers as well as with disease prognosis in larger independent cohorts of this malignancy will constitute the subject of future studies. However, this does not undermine the relevance of our study, which is the first to highlight the link between biotinidase and thyroid cancer and among the few that illustrate and strengthen the burgeoning evidence implicating biotinidase as a factor in cancer aggressiveness. Additional work in this area could shed crucial light on the mechanism of aggressiveness in thyroid cancer as well as aid the discovery of novel mechanism(s) that may account for the potential of biotinidase to determine aggressiveness of thyroid cancer. This valuable knowledge can give a significant boost to current research in the field by providing novel avenues for future work. Furthermore, the potential applicability that biotinidase presents as an aid to FNA diagnosis sets the stage for improving the management of aggressive papillary thyroid cancer and providing hope anew to patients and clinicians alike.
